# *WRINKLED1* transcription factor orchestrates the regulation of carbon partitioning for C18:1 (oleic acid) accumulation in Siberian apricot kernel

**DOI:** 10.1038/s41598-019-39236-9

**Published:** 2019-02-25

**Authors:** Shuya Deng, Yiting Mai, Lanya Shui, Jun Niu

**Affiliations:** Hainan Key Laboratory for Sustainable Utilization of Tropical Bioresource, Institute of Tropical Agriculture and Forestry, Hainan University, Haikou, Hainan 570228 China

## Abstract

WRINKLED1 (WRI1), an APETALA2 (AP2)-type transcription factor, has been shown to be required for the regulation of carbon partitioning into fatty acid (FA) synthesis in plant seeds. To our knowledge, the regulatory network of WRI1 remains unknown in *Prunus sibirica* kernel (PSK), a novel woody biodiesel feedstock in China. In this study, based on the transcriptional data from developing oilseeds of multiple plant species, we identified 161 *WRI1*-coexpressed genes using weighted gene co-expression network analysis (WGCNA). The major portion of *WRI1*-coexpressed genes was characterized to be involved in carbon partitioning and FA biosynthesis. Additionally, we detected the temporal patterns for oil content and FA compositions in developing PSK from two different germplasms (AS-85 and AS-86). The major differences between the two germplasms are higher contents of oil and C18:1 in AS-85 than in AS-86 at a mature stage. Thus, AS-85 and AS-86 are desirable materials to explore the molecular and metabolic mechanisms of oil accumulation in Siberian apricot. Expression analysis in developing PSK of AS-85 and AS-86 indicated that the expression level of *P*. *sibirica WRI1* (PsWRI1) was closely correlated to accumulative rate of oil. Also, the comparison of expression profiles in developing PSK of AS-85 and AS-86 displayed that the *pPK*, *E1-α*, *E2*, *TAL*, *BC*, *MCMT*, *BS*, *SAD* and *FAD2* have a high correlation with *PsWRI1*. Transient expression showed that Pro_SAD_- and Pro_BS_-driving GUS expression showed no substantial difference between AS-85 and AS-86, while the expression level of Pro_PEPCK_-AS-85 driving GUS was significantly higher than that of Pro_PEPCK_-AS-86 driving GUS. Additionally, transient co-transformation with *PsWRI1* revealed that Pro_SAD_, Pro_PEPCK_ and Pro_BS_ activity could be specifically up-regulated by PsWRI1. This regulatory mechanism of PsWRI1 may create a steep concentration difference, thereby facilitating carbon flux into C18:1 accumulation in developing PSK. Overall, all our findings imply a versatile mechanism of WRI1 to optimize carbon allocation for oil accumulation, which can provide reference for researching the woody biodiesel plants.

## Introduction

Siberian apricot (*Prunus sibirica* L.) belongs to the Rosaceae family. In China, the total area of Siberian apricot is approximately 1.7 million ha^1^ and the annual harvest of seeds is nearly 192,500 tons^2^. In the previous study, we have found that Siberian apricot kernel (PSK) had a high oil content (over 50%) which mainly comprised C18:1 (oleic acid) and C18:2 (linoleic acid)^2,3^. Moreover, according to the investigation of biodiesel fuel properties, such as cold filter plugging point, cetane number, oxidative stability and flash point, the PSK oil has been determined to be suitable for biodiesel production^4^. Thus, Siberian apricot has become a more critical component of woody oilseed species.

Generally, seed development is a highly controlled developmental program that can be divided into three main steps: morphogenesis, maturation and late maturation^5^. Several studies have reported that the accumulation of storage compounds (including protein, starch and lipid) usually occurs during the maturation stage^6,7^. Presently, plant seeds store lipid mainly in the form of triacylglycerol (TAG) that could be degraded to provide carbon and energy during germination and early seedling growth^8^. Additionally, plant-derived oils are a major food for humans, and are an important source of margarines, salad oils, lubricants and biodiesel^9^. For these reasons, the accumulative metabolism of plant oil has been subjected to intensive studies, and hundreds of oil-related genes have been identified to be involved in a series of enzymatic reactions occurring in several subcellular organelles^10,11^. Also, recent studies in Siberian apricot provide details on the very large number of regulatory enzymes, transcription factors (TFs) and microRNAs (miRNAs) responsible for oil biosynthesis and accumulation^1–3^.

The main source of carbon for oil biosynthesis in heterotrophic tissues is sucrose, which could be converted to acetyl-CoA by glycolysis, pentose phosphate pathway (PPP) and pyruvate dehydrogenase complex^12^. The acetyl-CoA is the main precursor for malonyl-CoA destined to *de novo* fatty acid (FA) biosynthesis in plastid^13^. Also, *de novo* FA synthesis requires stoichiometric amounts of ATP, NADPH, and NADH for each sequential addition of an acetyl unit to the growing chain of FA^14^. The generated FAs (C16 or 18) can be further elongated, desaturated or otherwise modified in the endoplasmic reticulum (ER), and then they can participate in TAG assembly for storage oil^2,12,13,15^. All these findings indicate that oil biosynthesis as a part of the seed maturation process is a highly controlled developmental program. Indeed, previous studies in *Arabidopsis* delineated a complex network of TFs that control a series of gene expressions for oil biosynthesis, for example ABSCISIC ACID INSENSITIVE3 and 4 (ABI3 and 4)^16^, LEAFY COTYLEDON1 and 2 (LEC1 and 2)^17^, FUSCA3 (FUS3)^18^, and WRINKLED1 (WRI1)^19^. Importantly, a loss-of-function mutation, *WRI1*, in *Arabidopsis* causes an approximately 80% reduction in oil content compared with the wild type^20^. WRI1, an APETALA2 (AP2)-type transcription factor, can activate genes via binding to the conserved AW-box, [CnTnG](n)7[CG] (n represents any nucleotide)^21^. In various plant species, it has been proved that WRI1 expression is pivotal in directing the carbon flux from glycolysis into FA biosynthesis^13,19–21^. Despite an increasing body of physiological, biochemical, genetic and molecular data in model plants, the regulatory role of *P*. *sibirica WRI1* (PsWRI1) in oil accumulation of PSK remains unclear.

Recently, advances in molecular and sequencing technology have made transcription patterns during oilseed development to become an effective choice for the identification of genes involved in oil biosynthesis and accumulation, such as Siberian apricot^2^, oil palm^12^ and castor^13^. Based on those transcriptomic data, the analysis of gene co-expression network has been feasible to identify functional module. Weighted gene co-expression network analysis (WGCNA), one of the most useful gene co-expression network based approaches, allows a global interpretation of gene expression data by constructing gene networks based on similarities in expression profiles among samples^22^. These coexpressed genes are likely to be functionally related, and may participate in similar biological processes^23^. Therefore, the analysis of gene co-expression network has been conducted to reconstruct regulatory pathways, discover novel candidate genes, and identify key modulators^23–26^.

To infer the biological roles of WRI1 in oil plants, we took advantage of 22 publicly available transcriptome databases derived from Siberian apricot, oil palm, castor, rapeseed and burning bush to study the coexpression genes with WRI1. Also, Gene Ontology (GO) and Kyoto Encyclopedia of Genes and Genomes (KEGG) were used to categorize the functions of WRI1-coexpressed genes. Furthermore, two different germplasms (AS-85 and AS-86) of Siberian apricot were used as experimental materials, and their oil contents and FA compositions were first detected at different developing stages. Subsequently, according to the WGCNA results, we conducted comparative expression analysis of *PsWRI1* and its coexpressed genes using quantitative reverse transcription PCR (qRT-PCR). Several *PsWRI1*-coexpressed genes were selected for promoter cloned and transient expression analysis. Also, co-transformation with *PsWRI1* was performed to verify whether the activity of the selected *PsWRI1*-coexpressed genes was specifically regulated by PsWRI1. This work can provide novel insights into the function of PsWRI1 in Siberian apricot for increasing oil production in seeds.

## Results

### Co-expression network analysis of *WRI1* gene

Based on previous transcriptome data from developing oilseeds of Siberian apricot^2^, oil palm^12^, castor, rapeseed and burning bush^13^, we screened and obtained 4011 shared genes with the same TAIR numbers, all of which expressed among the above species (Table S1). Thus, these genes may be considered to be constitutively transcribed in the 5 oilseeds. Subsequently, we attempted to establish the coexpression network using the R package of WGCNA. The WGCNA software is a comprehensive collection of R functions for performing various aspects of weighted correlation network analysis, including functions for network construction, module detection, gene selection, calculations of topological properties, data simulation^22^. By using a convenient one-step network construction of WGCNA, we extracted and ranked the 161 genes whose transcriptional levels most tightly correlated with *WRI1* (Table S2), implying that these *WRI1*-coexpressed genes may be under the same metabolic pathways.

### Functional classification of *WRI1*-coexpressed genes

**Figure 1:**
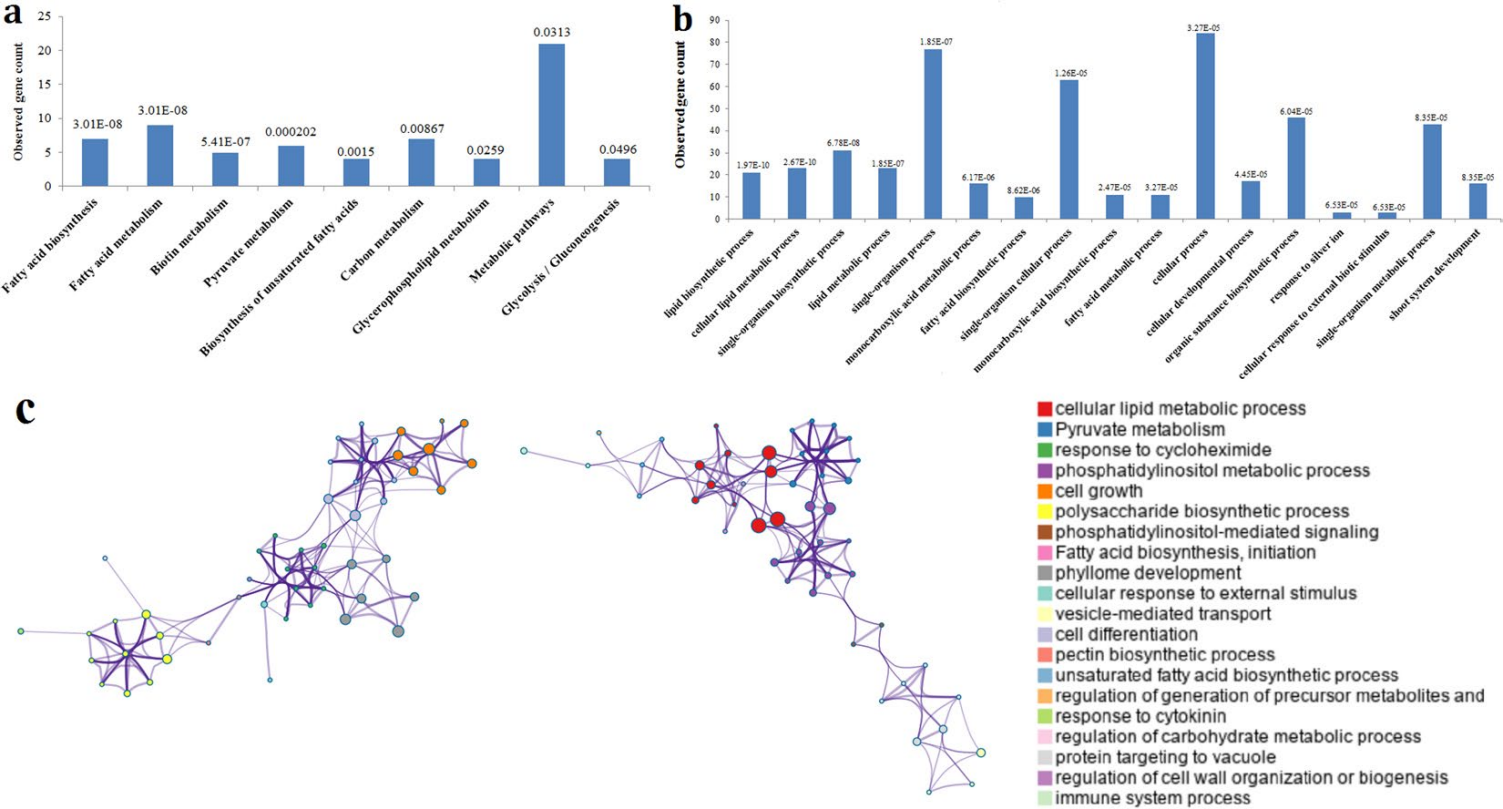
Functional clustering analysis of *WRI1*-corelated genes. (**a**) KEGG analysis of *WRI1*-coexpressed genes. (**b**) GO analysis of *WRI1*-coexpressed genes. These data labels on the histograms represent the FDR values. (**c**) Visual enrichment analysis by Metascape. The detailed results have been shown in Table S2.

A better understanding of the functions of *WRI1*-coexpressed genes will help us identify the biological function of WRI1. We performed KEGG and GO clustering, and found a high correlation among *WRI1*-coexpressed genes (Fig. 1). Such an enrichment indicates that *WRI1*-coexpressed genes are at least partially biologically connected in developing oilseeds. By KEGG analysis, support for this view came from a finding of *WRI1*-coexpressed genes enriched in FA biosynthesis and pyruvate metabolism in developing oilseeds (Fig. 1a). Also consistent with this result, *WRI1*-coexpressed genes were characterized to be credibly implicated in “GO.0008610 lipid biosynthetic process”, “GO.0044255 cellular lipid metabolic process” and “GO.0006629 lipid metabolic process” with low false discovery rate (FDR), indicating a reliable enrichment of *WRI1*-coexpressed genes in these biological processes (Fig. 1b,c). Intriguingly, KEGG analysis showed that some *WRI1*-coexpressed genes function in biotin metabolism (Fig. 1a). Together, our functional enrichment highlights multiple functions for WRI1 in carbon partitioning and FA biosynthesis, desaturation or other modifications.

### Dynamic changes of oil content and FA compositions in developing PSK

**Figure 2:**
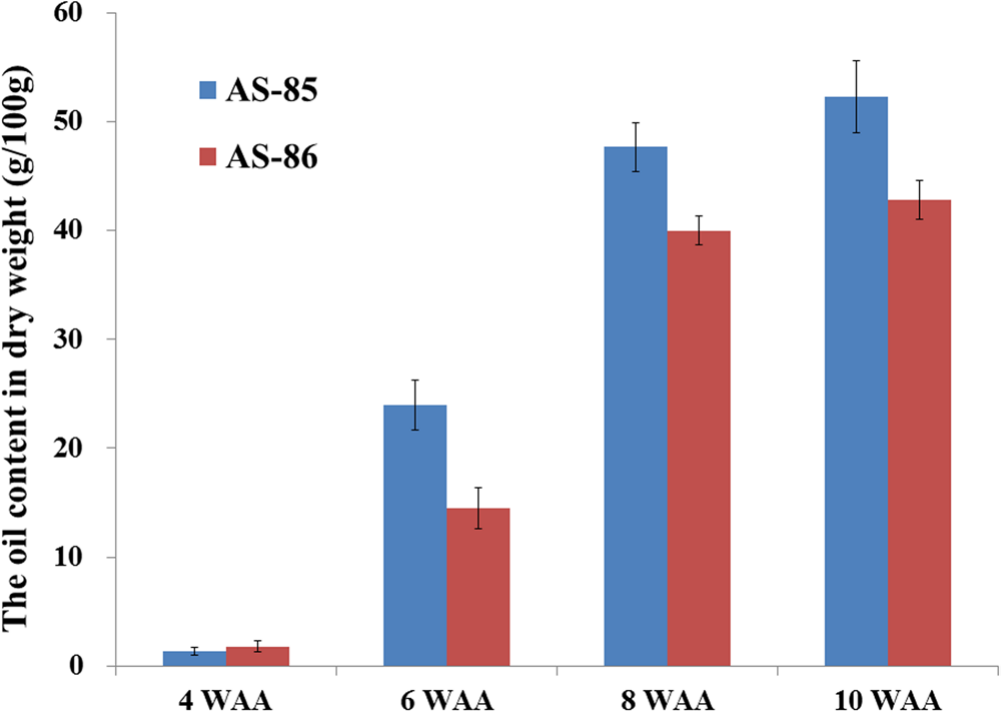
The PSK oil content by Soxhlet method at different development period. Data are means ± SE of three separate measurements.

In general, high oil content in oil plants is considered to be the vital metric for commercial development. The previous study has indicated that the accumulative pattern of PSK oil showed a sigmoid pattern during development stage^1^. Thus, the PSK samples of AS-85 and AS-86 at 4, 6, 8 and 10 weeks after anthesis (WAA) were selected as the experimental materials for comparative analysis. We characterized a gradual increase of PSK oil contents from 1.35 to 52.25 g/100 g and 1.81 to 42.79 g/100 g in AS-85 and AS-86, respectively (Fig. 2). Importantly, the oil increase of AS-85 (22.57 g) showed a higher accumulation than that of AS-86 (12.71 g) during 4–6 WAA, which ultimately led to the significant difference in total oil content between AS-85 and AS-86 (Fig. 2). By using GC-MS analysis, eight species of FAs were detected both in AS-85 and AS-86 (Table 1), as previously reported in PSK^1,4^. Among those FAs, C18:1 (oleic acid) and C18:2 (linoleic acid) represented the major compositions in PSK oil, and showed a remarkable elevation at 4–8 WAA (Table 1). Importantly, a major difference between the two germplasms was that C18:1 content was higher in AS-85 than in AS-86 at the last stage (Table 1). Together, the significant differences in the oil and C18:1 content between AS-85 and AS-86 indicate that the two germplasms would be desirable materials to explore the molecular and metabolic mechanisms of oil accumulation in *P*. *sibirica*.

**Table 1:** Changes in the FA composition by GC-MS during PSK development.

	AS-85 (g/100 g)				AS-86 (g/100 g)			
	4 WAA	6 WAA	8 WAA	10 WAA	4 WAA	6 WAA	8 WAA	10 WAA
C16:0	0.270 ± 0.029	1.200 ± 0.022	1.833 ± 0.050	2.017 ± 0.055	0.354 ± 0.006	0.784 ± 0.010	2.076 ± 0.031	1.897 ± 0.021
C16:1	0.013 ± 0.001	0.218 ± 0.015	0.281 ± 0.002	0.337 ± 0.005	—	0.093 ± 0.012	0.344 ± 0.014	0.314 ± 0.007
C18:0	0.033 ± 0.010	0.231 ± 0.006	0.486 ± 0.034	0.523 ± 0.031	0.076 ± 0.001	0.166 ± 0.016	0.505 ± 0.008	0.453 ± 0.001
C18:1	0.198 ± 0.013	12.773 ± 0.616	31.351 ± 0.647	35.912 ± 0.519	0.229 ± 0.012	7.152 ± 0.879	23.810 ± 0.218	26.987 ± 0.298
C18:2	0.786 ± 0.032	9.387 ± 0.087	13.526 ± 0.180	13.319 ± 0.330	1.002 ± 0.023	6.221 ± 1.001	13.123 ± 0.111	12.988 ± 0.008
C18:3	0.046 ± 0.004	0.066 ± 0.010	0.050 ± 0.013	0.031 ± 0.004	0.122 ± 0.019	0.061 ± 0.023	0.068 ± 0.006	0.087 ± 0.003
C20:0	—	0.026 ± 0.002	0.059 ± 0.013	0.044 ± 0.008	0.024 ± 0.009	0.023 ± 0.008	0.032 ± 0.002	0.033 ± 0.004
C20:1	—	0.021 ± 0.006	0.049 ± 0.003	0.047 ± 0.001	—	0.025 ± 0.005	0.030 ± 0.001	0.033 ± 0.007

### Early expression of *PsWRI1* gene contributing to oil accumulation in developing PSK

**Figure 3:**
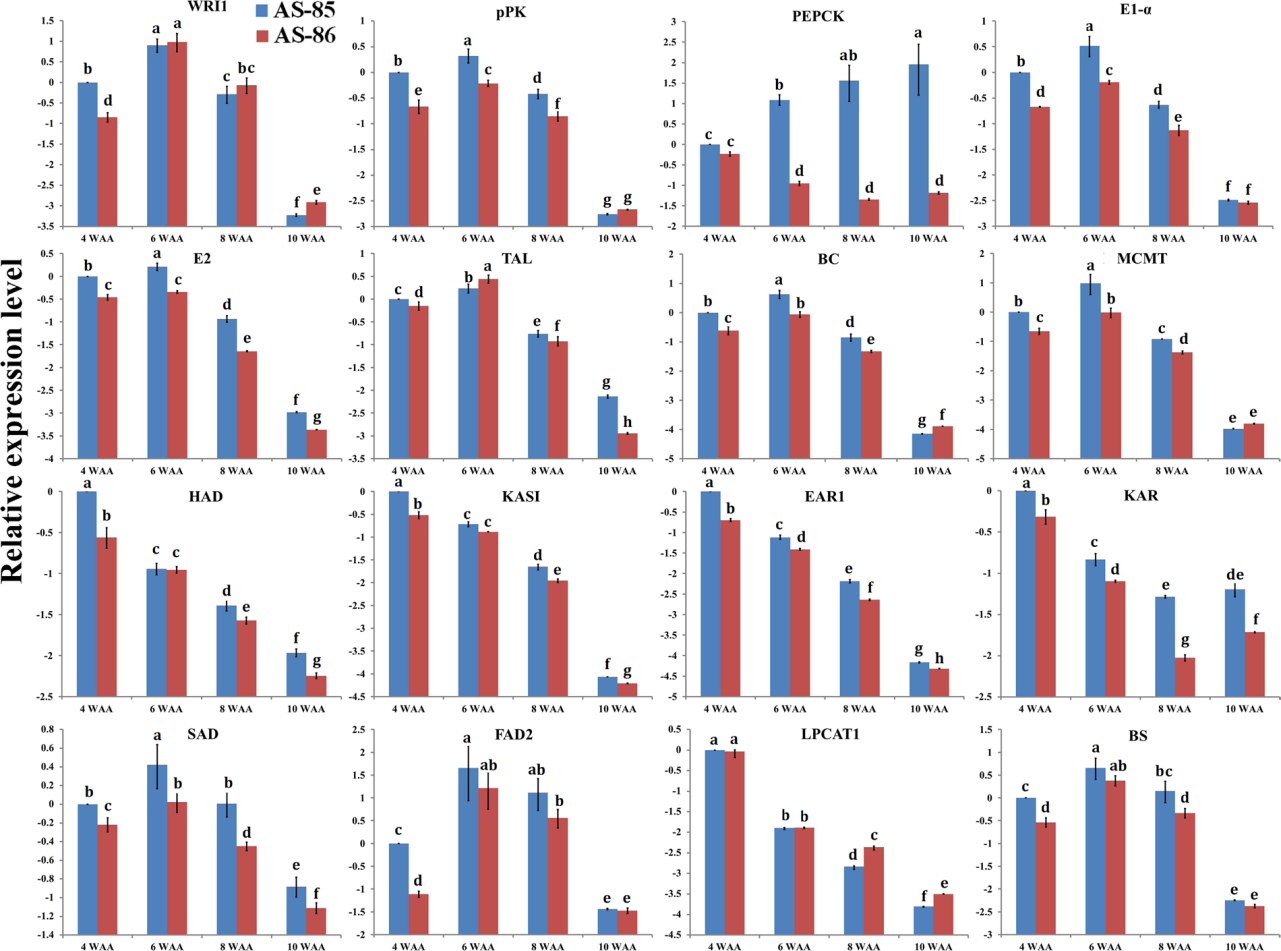
The qRT-PCR analysis of *WRI1* and its coexpression genes in developing PSK of AS-85 and AS-86. The ghecyclophilin and ubiquitin-conjugating enzyme were used as internal controls^49^, and the expression data of AS-85 at 4 WAA served as control. The relative expression levels were calculated as log_2_((1 + E1)^△Ct1(Control-Sample)^/(1 + E2)^△Ct2(Control-Sample)^), E1: PCR efficiency of target-gene primer; E2: PCR efficiency of reference-gene primer; △Ct1: the difference of Ct value between control and sample in experimental group; the difference of Ct value between control and sample in reference group. The significance of differences were analyzed using Duncan method (*p* < 0.01). Abbreviations: BC, biotin carboxylase; BS, biotin synthase; EAR1, enoyl-ACP reductase 1; FAD2, fatty acid desaturase 2; HAD, hydroxyacyl-ACP dehydrase; KAR, ketoacyl-ACP reductase; KAS, ketoacyl-ACP synthase; LPCAT, acyl-CoA: lysophosphatidylcholine acyltransferase; MCMT, malonyl-CoA: ACP malonyltransferase; PEPCK, phosphoenolpyruvate carboxykinase; pPK, plastidial pyruvate kinase; SAD, stearoyl-ACP desaturase; TAL, transaldolase; WRI1, WRINKLED1.

To investigate whether the *WRI1* (AT3G54320) expression has any influence on PSK oil accumulation, the expression pattern of *PsWRI1* gene during PSK development was analyzed by using qRT-PCR in AS-85 and AS-86 (Fig. 3). Expression data in AS-85 and AS-86 indicated that *PsWRI1* gene displayed a similarly bell-shaped pattern of expression (a maximal level at 6 WAA) during PSK development (Fig. 3). Intriguingly, the most striking difference with regard to the temporal pattern of *PsWRI1* expression was a higher transcription at 4 WAA in AS-85 than in AS-86 (Fig. 3). However, the expression levels for *PsWRI1* gene at other periods showed no substantial difference between AS-85 and AS-86 (Fig. 3). Our data imply, as noted above for a higher accumulative rate of oil in 4–6 WAA of AS-85 (Fig. 2), that the expression level of *PsWRI1* was closely correlated to accumulative rate of oil in PSK development.

### *PsWRI1*-mediated output of carbon flux in glycolysis during PSK development

The pyruvate required for oil biosynthesis is mostly from glycolysis, in which pyruvate kinase (PK) catalyzes the last conversion of phosphoenolpyruvate (PEP) to pyruvate in both cytosol (cPK) and plastid (pPK; AT5G52920)^12^. Aside from glycolysis, PEP also can be generated by PEP carboxykinase (PEPCK; AT4G37870)^27^. Our coexpression analysis characterized a coordinated expression of *pPK* and *PEPCK* with *WRI* (Table S3). However, qRT-PCR showed that only *pPK* had a consistent trend of the relative expression with *WRI* (*p* < 0.01) in developing PSK of AS-85 and AS-86, whereas *PEPCK* displayed a gradual increase and decrease in AS-85 and AS-86, respectively (Fig. 3).

Transaldolase (TAL; AT1G12230) is important for the balance of metabolites in the PPP, and is responsible for the generation of glyceraldehyde 3-phosphate (GAP) for plastidial glycolysis^28,29^. In this study, the expression levels of plastidial TAL were comparable to that of PsWRI1 (*p* < 0.01) (Fig. 3), and showed a correlation with oil synthesis (Fig. 2). Our results imply that PsWRI1 may play a major role in regulation of plastidial PPP for high rates of FA synthesis in PSK development.

To our knowledge, plastidial pyruvate dehydrogenase complex (PDC), including E1-α, E1-β, E2, and E3 subunit, is thought to be implicated in the conversion of pyruvate to acetyl-CoA for *de novo* FA biosynthesis in plants^7^. Coexpression analysis characterized E1-α (AT1G01090) and E2 (AT1G34430) subunit with *WRI1*-coexpressed genes (Table S3), and indeed a highly correlated temporal expression pattern (*p* < 0.05) was identified by qRT-PCR in developing PSK of AS-85 and AS-86 (Fig. 3). Also, we observed the higher expression levels for *E1-α* and *E2* in AS-85 than in AS-86 (Fig. 3). These results suggest, as noted for *pPK*, that PsWRI1 may be responsible for output rather than input in carbon flux to generate more carbon source for acetyl-CoA biosynthesis.

### *PsWRI1*-mediated input of carbon flux in FA biosynthesis during PSK development

A multi-subunit acetyl-CoA carboxylase (ACC) complex, including biotin carboxylase (BC; AT5G35360), biotin carboxyl carrier protein (BCCP) and carboxyltransferase (CT), catalyzes the carboxylation of acetyl-CoA to malonyl-CoA^30^. Subsequently, malonyl-CoA is transferred to malonyl-ACP by malonyl-CoA: ACP malonyltransferase (MCMT; AT2G30200) to provide two-carbon unit at each step of elongation^31^. There are four reactions of condensation, reduction, dehydration and reduction occurring in each synthesized cycle, which is catalyzed by a series of regulatory enzymes, including ketoacyl-ACP reductase (KAR; AT1G24360), hydroxyacyl-ACP dehydrase (HAD; AT5G10160), enoyl-ACP reductase (EAR; AT2G05990), ketoacyl-ACP Synthase (KAS; AT5G46290)^11^. An intriguing observation was that most of enzymes (BC, MCMT, HAD, KASI, EAR1 and KAR) were identified as *WRI1*-coexpressed genes in the present work (Table S3), implying a wide involvement of WRI1 targets in FA biosynthesis. In developing PSK of AS-85 and AS-86, the temporal expression of *BC* and *MCMT* closely matched the pattern of *PsWRI1* (*p* < 0.01) (Fig. 3), which may reflect a major contribution of PsWRI1 in developing PSK by controlling influx of acetyl-CoA to FA biosynthesis. However, the other FA synthases showed a coordinated down-expression in developing PSK (Fig. 3). As expected, most enzymes involved in FA biosynthesis showed a higher expression level in AS-85 than in AS-86 (Fig. 3).

### Biotin biosynthesis

It was intriguing that *WRI1*-coexpressed genes were characterized to be credibly implicated in biotin biosynthesis (Fig. 1a). Furthermore, biotin synthase (BS; AT2G43360) that catalyzes the conversion of dethiobiotin to biotin has been identified with a consistent trend of the relative expression with *PsWRI1* gene (*p* < 0.01) in developing PSK of AS-85 and AS-86 (Fig. 3). In addition, the expression levels of *BS* gene were higher in AS-85, especially at 4WAA, relative to AS-86 (Fig. 3). These results suggest that PsWRI1 appears to be intimately involved in the transcriptional regulation of *BS* gene.

### The C18:1 biosynthesis in developing PSK

After 7 elongation cycles, some produced 16:0-ACP are released from the FA biosynthesis, whereas others are elongated to 18:0-ACP by KASII and efficiently desaturated to 18:1-ACP by a stearoyl-ACP desaturase (SAD; AT2G43710)^32^. One *WRI1*-coexpressed gene was identified as a homologous gene of *SAD*, which exhibited a similarly bell-shaped expression pattern (a maximal level at 6 WAA; *p* < 0.01) in developing PSK of AS-85 and AS-86 (Fig. 3). Compared with AS-86, AS-85 had a higher expression of *SAD* gene (Fig. 3), which could explain the faster increasing trend of C18:1 (oleic acid) content in AS-85 (Table 1). Thus, we consider that the close correlation between *PsWRI1* and *SAD* expression in developing PSK suggests the regulatory role of PsWRI1 in C18:1 accumulation, leading to an ongoing outflow of FA to monounsaturated FA.

### Limiting factor with regard to the synthesis of polyunsaturated FAs in developing PSK

The synthesized FAs should be exported from the plastid to ER for further esterification or modification^11^. For the synthesis of polyunsaturated FAs, the C18:1 must be esterified by acyl-CoA: lysophosphatidylcholine acyltransferase (LPCAT; AT1G12640) to phosphatidylcholine (PC) pool in ER, and then desaturated by FA desaturase 2 (FAD2; AT3G12120) in PC pool^33^. Comparative analysis of expression profiles between AS-85 and AS-86 indicated that *FAD2* gene showed an expression relation with *PsWRI1* (*p* < 0.01), and had a higher level in AS-85 than in AS-86 (Fig. 3). Unexpectedly, there was no obvious difference in C18:2 accumulation between AS-85 and AS-86 (Table 1). Considering the important role of LPCAT1 in transport of newly synthesized FAs to PC for further desaturation in developing PSK^2^, we detected the expression level of *LPCAT1* gene that was not the WRI1-coexpressed genes in this study. Interestingly, the expression levels for *LPCAT1* displayed a similar profile during PSK development between AS-85 and AS-86 (Fig. 3). These comparisons strongly suggest LPCAT-mediated FA flux rather than transcriptional regulation of FAD2 as a major factor associated with the accumulation of polyunsaturated FAs in PSK development.

### Transient expression of promoter-GUS in *Nicotiana benthamiana*

**Figure 4:**
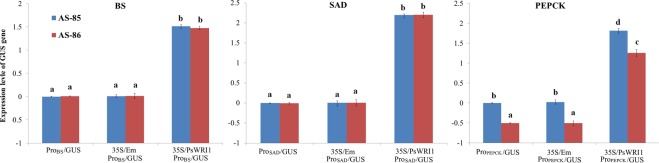
Comparative analysis of GUS transcription by the *Agrobacterium*-mediated transient expression system in *Nicotiana benthamiana*. Pro_(name)_/GUS means GUS transcription driven by the gene promoter. Also, Pro_(name)_/GUS was co-transformed with 35 S/PsWRI1 and 35 S/Em (control), respectively. The transcript level of GUS was determined by qRT-PCR using the actin and ubiquitin as an endogenous reference. The significance of differences were analyzed using Duncan method (*p* < 0.01).

Among these detected genes, only *PEPCK* showed a different expression pattern between AS-85 and AS-86 (Fig. 3). Also, the involvement of WR11 in *BS* transcriptional regulation remains unclear, and SAD has an important role in C18:1 accumulation. Thus, the three gene promoters were cloned from AS-85 and AS-86, and named as Pro_PEPCK_-AS-85, Pro_PEPCK_-AS-86, Pro_BS_-AS-85, Pro_BS_-AS-86, Pro_SAD_-AS-85 and Pro_SAD_-AS-86, respectively. Motif analysis indicated that Pro_SAD_ and Pro_BS_ had the same *cis*-acting elements between AS-85 and AS-86 (Table S3). However, in contrast to Pro_PEPCK_-AS-86, Pro_PEPCK_-AS-85 specifically contained the binding sites of calmodulin-binding transcription activator 3 (VCGCGB, −241), ABA INSENSITIVE 3 (CATGCA, −319 and −317) and Dof-type zinc finger transcription factor (TAAAG, −585 and −590) (Table S3). To investigate whether the promoter has any influence on gene expression, transient expression carrying promoter-GUS chimeric vectors were performed (Fig. 4). Expectedly, we observed that Pro_SAD_- and Pro_BS_-driving GUS expression showed no substantial difference between AS-85 and AS-86 (Fig. 4). However, the expression level for GUS was significant higher in Pro_PEPCK_-AS-85/GUS than in Pro_PEPCK_-AS-86/GUS (Fig. 4), indicating that the promoter differences could result in the PEPCK expression difference.

WRI1 activates genes via binding to AW-box target sites containing the sequence [CnTnG](n)7[CG], where n represents any nucleotide^21^. To explore whether the activity of Pro_SAD,_ Pro_PEPCK_ and Pro_BS_ was specifically regulated by PsWRI1, we constructed *PsWRI1* plant expression vector (35 S/PsWRI1) as effector, and meanwhile various promoter-GUS chimeric vectors were used as reporters. Transient co-transformation of *Nicotiana benthamiana* indicated that Pro_SAD_-, Pro_PEPCK_- and Pro_BS_-driving GUS expression was significantly increased compared with the control (35 S/Em Pro_PEPCK_/GUS) (Fig. 4), suggesting that Pro_SAD,_ Pro_PEPCK_ and Pro_BS_ activity could be specifically up-regulated by PsWRI1. Indeed, characterization of *cis*-regulatory elements revealed that the Pro_SAD,_ Pro_BS_ and Pro_PEPCK_ sequences had the core binding *cis*-element of WRI1 (Table S3).

## Discussion

**Figure 5:**
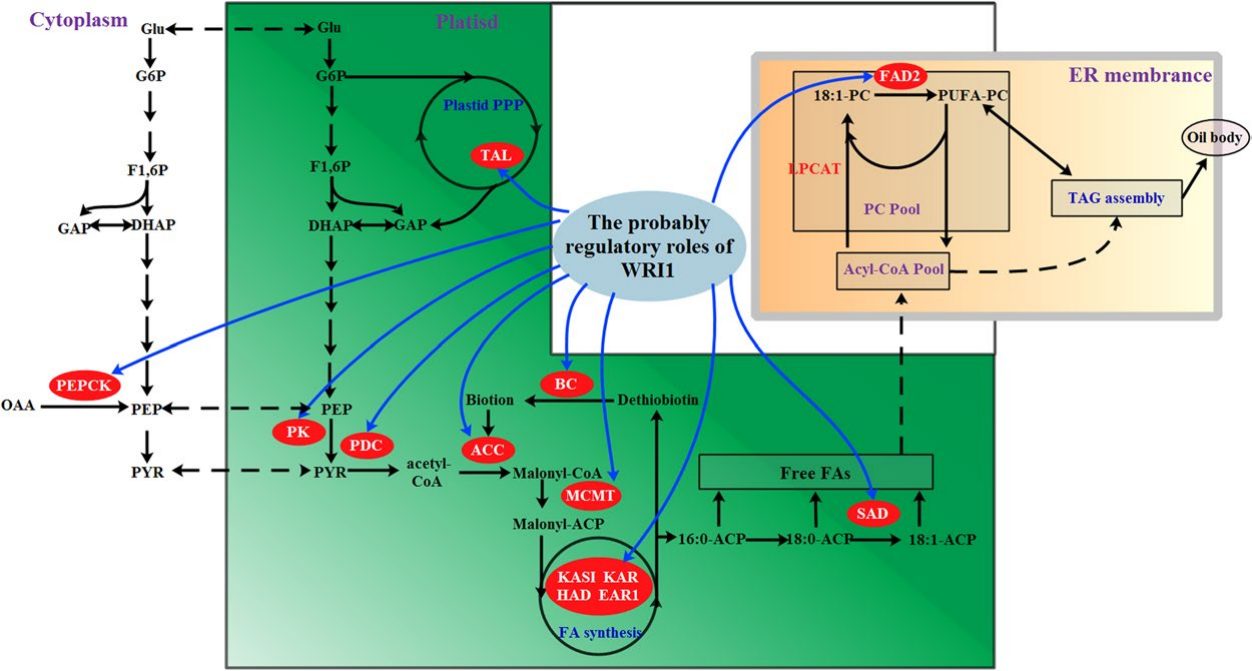
Proposed model of PsWRI1 regulation in PSK oil accumulation. Blue arrow lines represent the possible transcription regulation sites of PsWRI1. Abbreviations: BC, biotin carboxylase; BS, biotin synthase; EAR1, enoyl-ACP reductase 1; FAD2, fatty acid desaturase 2; G6P, Glucose-6-phosphate; Glu, glucose; HAD, hydroxyacyl-ACP dehydrase; KAR, ketoacyl-ACP reductase; KAS, ketoacyl-ACP synthase; LPCAT, acyl-CoA: lysophosphatidylcholine acyltransferase; MCMT, malonyl-CoA: ACP malonyltransferase; PEP, phosphoenolpyruvate; PEPCK, phosphoenolpyruvate carboxykinase; pPK, plastidial pyruvate kinase; PYR, pyruvate; SAD, stearoyl-ACP desaturase; TAL, transaldolase; OAA, oxaloacetate; pPK, plastidial pyruvate kinase; PYR, pyruvate.

Despite many TFs such as ABI3, LEC1, LEC2 and FUS3 were identified to be responsible for oil synthesis and deposition, only *WRI1* gene was highly up-regulated in oil palm compared to date palm^12^, and its expression has been found to be closely connected to oil accumulation in various plants^19–21,34^. However, the biological role of WRI1 in PSK oil accumulation remains elusive. On the basis of this theory that TF and its direct target genes may have similar expression patterns^22,25,35^, we utilized 22 publicly available transcriptome databases to identify 161 *WRI1*-coexpressed genes by the R package of WGCNA. Many studies in *Arabidopsis* elucidated a number of WRI1-targeted genes encoding enzymes responsible for glycolysis, FA synthesis and TAG assembly^20,34^. By KEGG and GO analysis, we characterized most of *WRI1*-coexpressed genes to be implicated to pyruvate metabolism, lipid metabolism and FA modification, whereas no *WRI1*-coexpressed genes were identified to be involved in TAG assembly. An intriguing observation is that the temporal patterns of gene expression for the enzymes of FA synthesis and for TAG assembly are very different during seed development^11^. Thus, this difference may contribute to low transcriptional correlation of genes in TAG assembly with *WRI1* gene. From this WRI1 regulatory network (Fig. 5), 14 genes encoding key enzymes implicated in carbon partitioning, FA biosynthesis and desaturation were further investigated in this present work. By the transient expression in *N*. *benthamiana*, Pro_SAD_-, Pro_PEPCK_- and Pro_BS_-driving GUS expressions were enhanced when co-transformed with 35 S/PsWRI1 (Fig. 4), indicating that PsWRI1 could transcriptional regulate SAD, PEPCK and BS expression. Our results demonstrate that WGCNA analysis of *WRI1*-coexpressed genes is feasible.

Generally, transformation of foreign gene into plant is highly efficient manner of studying gene function. However, owing to lack of the technology for tissue regeneration in Siberian apricot, we used two different Siberian Apricot germplasms to explore the biological role of WRI1 in PSK oil accumulation. Based on the previous investigation on developing kernel^1^ and collection of Siberian Apricot germplasms^4^, the PSKs of AS-85 and AS-86 at 4, 6, 8 and 10 WAA were selected for comparative analysis of oil content, FA composition, and gene expression. A gene expression map of *Arabidopsis thaliana* indicated many core genes involved in FA synthesis display a bell-shaped pattern of expression during seed development^36^. Indeed, most genes such as *WRI1*, *pPK*, *TAL*, *E1-α*, *E2*, *BC*, *MCMT*, *SAD* and *FAD2* showed a bell-shaped pattern of expression (a maximal level at 6 WAA) during PSK development of AS-85 and AS-86 (Fig. 3).

In previous reports, such as *Arabidopsis*^34^, maize^35^, rapeseed^8^ and potato^19^, it has been proved that overexpression of *WRI1* could lead to an significant increase in oil content. In this study, *PsWRI1* displayed high expression levels during 4–8 WAA both in AS-85 and AS-86, and the *PsWRI1* expression of AS-85 had a higher level than that of AS-86 at 4 WAA (Fig. 3). In addition, we observed a major period of oil accumulation of during 4–8 WAA, and a higher accumulative rate of oil at 4–6 WAA in AS-85, relative to AS-86 (Fig. 2). Summarily, these results indicated the expression level of *PsWRI1* may be closely related to accumulative rate of PSK oil, suggesting the pivotal role of PsWRI1 central to the oil production.

The PPP is thought to not only provide plants with important substrates for metabolism, but also generate large amounts of reducing power to drive various anabolic processes^28^. In the PPP, TAL is important for the balance of metabolites and is involved in the glyceraldehyde 3-phosphate (GAP) production for glycolysis^28,29^. The carbon source (GAP) and reducing power (NADPH) generated by PPP both could be used for FA biosynthesis^31^. Interestingly, the expression profile of *TAL* gene displayed a similarly bell-shaped pattern with that of *PsWRI1* gene (Fig. 3), as has been reported for developing PSK by transcriptome analysis^2^. Thus, PsWRI1 may be involved in the regulation of PPP to generate reducing power for FA biosynthesis in PSK development. A major flux through glycolysis is expected to provide the large amounts of pyruvate required for high oil synthesis in oilseeds^13^. As the rate-limiting enzyme, PK catalyzes the last conversion of PEP to pyruvate^12^. Our qRT-PCR imply that pPK may be directly regulated by PsWRI1 in PSK, as was reported in *Arabidopsis*^20^. In addition, our data implicate transcriptional regulation of pPK rather than of cPK as a major factor associated with the production of pyruvate via glycolysis for FA biosynthesis in PSK development (Fig. 5), which is consistent with our previous conclusion that pPK plays a major role in providing pyruvate for FA synthesis in PSK development^2^.

Besides of glycolysis, PEPCK in the presence of oxaloacetate can provide PEP for pyruvate generation^27^. The qRT-PCR analysis indicated that *PEPCK* showed an up- and down-regulation in AS-85 and AS-86, respectively (Fig. 3). In addition, the previous transcriptome analysis in PSK development revealed that *PEPCK* gene displayed an U-shape expression pattern, a minimum value at early-middle development^2^. One possible reason for the various expression patterns of the *PEPCK* gene is the difference in the promoter sequence. Motif analysis of Pro_PEPCK_ indicated Pro_PEPCK_-AS-85 specifically contained the *cis*-acting elements of calmodulin-binding transcription activator 3 (VCGCGB, −241), ABA INSENSITIVE 3 (CATGCA, −319 and −317) and Dof-type zinc finger transcription factor (TAAAG, −585 and −590) (Table S3). This result, combined with a differential expression of GUS between Pro_PEPCK_-AS-85/GUS and Pro_PEPCK_-AS-86/GUS (Fig. 4), implied that other TFs (ABI3) and signal factors (Ca^+^) may regulate the *PEPCK* transcription, leading to different expression pattern in different Siberian Apricot germplasms. As noted for the previous report, PEPCK is mainly involved in amino acid catabolism and dissimilation of organic acids in developing seeds^37^. Thus, we believe that PEPCK may not participate in providing carbon for FA biosynthesis in PSK development.

The acetyl-CoA required for *de novo* FA synthesis is provided by the activity of plastid PDC^7^. The expression pattern of *E1-α* and *E2* showed a high correlation with that of *PsWRI1* (Fig. 3), a bell-shaped pattern that was consistent with our previous transcriptome studies in developing PSK^2^. It is interesting to note that pPK and PDC are at the bottom of carbon flux in glycolysis (Fig. 5), implying that the PsWRI1 in developing PSK may regulate the output of carbon flux, which is important for achieving high oil yield^10^.

Carboxylation of acetyl-CoA to malonyl-CoA is the first committed step in FA synthesis and is catalyzed by a multisubunit ACC complex, including BC, BCCP and CT^30^. The biotin (vitamin H) has been recognized as a carrier of carbon dioxide, and is the essential cofactor of biotin-dependent carboxylases, such as pyruvate carboxylase, acetyl-CoA carboxylase and ACC^38^. It is well-known that a series of plastidial proteins (MCMT, HAD, KAS, EAR1 and KAR) are responsible for conversion of malonyl-CoA to FAs^11^. In agreement with the previous assumption that many genes from FA synthesis in *Arabidopsis* are the targets of WRI1^39^, most of genes involved in FA synthesis were identified as WRI1-coexpressed genes in this study (Table S2). Notably, the expression profiles of *BC*, *BS* and *MCMT* closely matched the bell-shaped pattern of *PsWRI1* expression, but *HAD*, *KASI*, *EAR1* and *KAR* showed a gradual down-expression in developing PSK (Fig. 4), similar to our previous transcriptome data in developing PSK^2^. Therefore, there may be an intricate regulatory network at transcriptional and post-transcriptional level controlling FA biosynthesis in developing PSK. The components of this network are largely unknown and, therefore, further investigation will be needed to understand metabolic networks involved in oil biosynthesis and their regulations. The most interesting finding was noted for the involvement of WR11 in *BS* transcriptional regulation, which has not been reported in previous studies. Our results suggested that *PsWRI1* expression could up-regulate BS transcription, which could promote carbon flow from glycolysis into FA biosynthesis.

After *de novo* FA synthesis, the saturated FAs could be further desaturated to monounsaturated and polyunsaturated FAs by SAD and FAD2, respectively^32,33^. Previous transcriptome study in developing PSK has found the shared expression profiles of *SAD*, *FAD2* and *WRI1*^2^. Indeed, our qRT-PCR confirmed that *SAD* and *FAD2* showed an expression correlation with *PsWRI1* (Fig. 3). Transient co-transformation indicated that Pro_SAD_ activity could be up-regulated by PsWRI1 (Fig. 4). Thus, these results lead us to speculate that WRI1 may participate in production of unsaturated FAs by controlling the expression of *SAD* and *FAD2* gene. Also consistent with this inference, expression analysis revealed that *SAD* gene showed a significantly higher expression in AS-85 compared with AS-86 (Fig. 4). Combined with our observation on a higher content of C18:1 in AS-85 than that in AS-86 (Table 1), we concluded that PsWRI1 has a primary role in promoting C18:1 accumulation in PSK, as was shown in *Arabidopsis* that seeds of the *WRI1* mutants showed a 10% reduction in C18:1 content^20^. In sharp contrast to the content of C18:1, the content of C18:2 showed no substantial difference between AS-85 and AS-86 (Table 1). However, *FAD2* as the *WRI1*-coexpressed gene showed a significant up-regulation at AS-85, implying that WRI1-mediated transcriptional regulation of FAD2 may not be the limiting factor for C18:2 accumulation in PSK. Given that the LPCAT is responsible for PC-deacylation half of the acyl editing cycle^11^ and important role of LPCAT1 in PSK^2^, the expression level of *LPCAT1* gene was analyzed. The similar expression levels of *LPCAT1* gene between AS-85 and AS-86 strongly implicate LPCAT-mediated FA flux with the accumulation of polyunsaturated FAs in developing PSK.

## Conclusions

We systematically and comprehensively analyzed the *WRI1*-coexpressed genes in five oil plants, and identified most coexpressed genes to be involved in carbon partitioning and FA biosynthesis, desaturation or other modifications. From this WRI1 regulatory network, many rate-limiting genes involved in glycolysis, PPP, and FA synthesis and desaturation, such as *pPK*, *TAL*, *E1-α*, *E2*, *BC*, *BS*, *MCMT*, *SAD* and *FAD2*, showed a high expression correlation with *PsWRI1* in developing PSK of AS-85 and As-86. Moreover, the expression level of *PsWRI1* and the accumulative rate of PSK oil were closely related in developing PSK of AS-85 and As-86. This would imply that PsWRI1 could control carbon flow into FA biosynthesis, especially into C18:1 accumulation, by transcriptional regulation of some core genes. Although no definitive answer can be provided at this time, Pro_SAD,_ Pro_PEPCK_ and Pro_BS_ activity was identified to be up-regulated by PsWRI1 using transient co-transformation in *N*. *benthamiana*, indicating the reliability of WGCNA in this study. This study will provide a fundamental basis for in-depth experimental studies of WRI1 function in *P*. *sibirica* for increasing oil production in seeds.

## Methods

### Plant material

Based on the previous collection of Siberian Apricot germplasms^4^, the kernels of two germplasms were identified with obvious differences in oil content, and were named AS-85 and AS-86, respectively. AS-85 and AS-86 located at the Beijing Forestry University experimental station, Beijing, China. Flowers with the same anthesis were marked, and then seeds were harvested at 4, 6, 8 and 10 WAA on the basis of our previous investigation on kernel characteristics, oil contents and FA compositions^1^. The oil content and FA composition should have obvious alteration during the four developmental stages. After removing the sarcocarp, the seeds were immediately frozen in liquid nitrogen and stored at −80 °C until use.

### PSK oil extraction and trans-esterification

The kernels were dried using an oven at 80 °C for 8–10 h and crushed. Subsequently, the PSK oils were extracted by n-hexane double extraction at 25 °C using a rotary evaporator (LABORTA 4000-efficient, Heidolph, Germany). The oil content was determined by the difference in weight of the dried kernel sample before and after the extraction. The determination was performed in triplicate.

Siberian apricot kernel oils were methylated following a trans-esterification method as Wang (2012) described^40^. The KOH (1 mass % with respect to oil) was dissolved in anhydrous methanol (5.5:1 mol ratio of methanol/oil). The methanol-KOH solution was added to the pre-heated PSK oil and stirred at 60–65 °C for 1 h. After cooling and standing, the reaction mixture was separated into two layers. The upper layer, including the resulting methyl esters, was isolated, dried with Na_2_SO_4_ and analyzed by gas chromatography to determine the FA composition.

### Fatty acid methyl ester analysis

The qualitative and quantitative analyses of obtained FA methyl esters were performed by GC-MS using the Agilent 6890 equipped with a flame ionization detector (GB/T17377-1998). The following chromatographic conditions were applied: inlet temperature 250 °C, detector temperature 280 °C and injection volume 1 µL. The carrier gas was high-purity hydrogen. Peak integration was performed by applying HP3398A software. The determination was run in triplicate.

### Coexpression analysis and functional annotation

To precisely pinpoint *WRI*-coexpressed genes, the raw data were downloaded from NCBI Short Read Archive database, including SRR1564517, SRR1568273, SRR1568275, SRR1568789 and SRR1568805 for Siberian apricot, SRX059258, SRX059259, SRX059260, SRX059261 and SRX059262 for oil palm, SRX090767, SRX090768, SRX090769 and SRX090771 for rapeseed, SRX007402, SRX007403, SRX007404, SRX007405 and SRX007406 for castor, and SRX070806, SRX029139, SRX029140, SRX029141, SRX029142 and SRX029135 for burning bush. Based on assembly results of cDNA library and annotated results against TAIR database^2,12,13^, the homologous TAIR locus with the maximum scorer and minimum E-value (an cut-off of 10^−5^) was assigned to the corresponding gene to sufficiently normalize these genes. Subsequently, the shared genes with the same TAIR number were selected from the above species for coexpression analysis. After removal of the low quality reads (including adapter sequences, reads containing ploy-N, and reads with more than 10% Q < 20 bases), the clean reads from different transcriptome databases were mapped to the shared genes in different cDNA libraries, respectively. The expression levels of genes were estimated using Fragment Per Kilobase of exon model per Million mapped reads (FPKM) by RSEM software^41^.

Using the R package of WGCNA^22^, automatic construction of the gene network and identification of modules were conducted according to the manual (labs.genetics.ucla.edu/horvath/CoexpressionNetwork/Rpackages/WGCNA/#manualInstall). To optimize data and avoid zero value, the values of Log_2_(FPKM + 0.001) were used for WGCNA (Supplementary Table S1). Using the data of Supplementary Table S1, the power 9 was selected by pickSoftThreshold analysis to amplify the strong connections between genes and penalize the weaker connections. Then, we used a convenient one-step network construction and module detection, and chose a relatively large minimum module size of 30 and a medium sensitivity (DeepSplit = 2) to cluster splitting. The WGCNA package also provides a convenient function for exporting the network to text format^22^. The WRI1-coexpressed genes were selected with threshold weight ≥ 0.1. The GO and KEGG analysis were performed by Blast2GO^42^ and KOBAS^43^ software, respectively. The FDR was used for the multiple testing of KEGG and GO enrichment^44^. Also, WRI1-coexpressed genes were analyzed by Metascape (metascape.org/gp/index.html#/main/step1) and statistically enriched using Cytoscape^45^.

### Transient expression and co-transformation in *Nicotiana benthamiana*

The gDNA from AS-85 and AS-86 was extracted using plant genome DNA extraction kit (Bioteke). Total RNA was separately extracted from the mixture using RNeasy Plant Mini Kits (Qiagen) according to the manufacturer’s protocol. Three biological repetitions were performed for each RNA extraction. The first-strand cDNA was synthesized by using oligo d(T) primers and reverse transcription System (Promega). The promoters (−1 kb upstream of the predicted translation start site) of the *WRI1*-coexpressed genes were cloned. The reverse primers were designed in the 5´ sequence based on our previous cDNA library^2^, while the forward primers were designed in conserved region based on the genome information of *P*. *persica*, *P*. *avium* and *P*. *mume* (www.ncbi.nlm.nih.gov/genome/gdv/), belonging to the same family Rosaceae with our experimental material. The primer sequences were shown in Supplementary Table S4. The promoter sequences were sequenced by Sangon (Guangzhou, China). Applications in the web-based promoter analysis program Motif Matcher (users.soe.ucsc.edu/~kent/improbizer/motifMatcher.html) and PLACE (www.dna.affrc.go.jp/PLACE/signalup.html) were used to analyze the *cis*-acting elements. According the ClonExpress® II One Step Cloning Kit (Vazyme), the promoters were inserted into the EcoRI and NcoI sites of vector pCAMBIA1301. The full-length cDNA sequence (*PsWRI1*) was amplified from PSK cDNA by PCR using two gene-specific primers: 5′-TCCTCTTCTTCTTCTTGGGTCTCTG-3′ (forward prime) and 5′-CACCCACATCAAATCACAACAAAC-3′ (reverse primer), and then cloned into pGM-T Vector (TIANGEN). A HindIII-EcoRI fragment from pGM-PsWRI1 containing the *PsWRI1* sequence was linked to the HindIII and EcoRI sites of pGreen0029 vector. For a negative control, empty effector plasmid 35 S/Em was constructed by the replacement of *PsWRI1* gene in pGreen0029. These constructs were introduced into *Agrobacterium tumefaciens* strain EHA105 via the freezing-thaw. *N*. *benthamiana* was transformed and co-transformed using a standard leaf-disc co-cultivation procedure^46–48^.

### Preparation of RNA and qRT-PCR

The amplification primers were designed using PrimerQuest (www.idtdna.com/PrimerQuest/Home/Index) software with melting temperatures at 62 °C, and the absence of secondary structures was verified by the UNAFold program (eu.idtdna.com/UNAFold). The qRT-PCR was performed using the SYBR Premix Ex Taq Kit (TaKaRa) according to the manufacturer’s protocol. Negative controls consisting of nuclease-free water instead of template, and reverse transcriptase controls prepared by substituting reverse transcriptase for nuclease-free water in the cDNA synthesis step were included in all analyses for each primer pair. Three technical repetitions were performed for qRT-PCR. According to our previous studies, cyclophilin and ubiquitin-conjugating enzyme were used as reference genes in PSK^49^, actin and ubiquitin were used as reference genes in *N*. *benthamiana*^48^. The qRT-PCR primer sequences were shown in Supplementary Table S4. The relative expression levels were calculated as log_2_((1 + E1)^ΔCt1(Control-Sample)^/(1 + E2)^ΔCt2(Control-Sample)^), E1: PCR efficiency of target-gene primer; E2: PCR efficiency of reference-gene primer; ΔCt1: the difference of Ct value between control and sample in experimental group; the difference of Ct value between control and sample in reference group. The PCR efficiency (E) was estimated from the data obtained from the exponential phase of each individual amplification plot and the equation (E = 10^slope^)^50^. The statistical analysis of expressed data was performed using SPSS software.

## Supplementary information

Supplementary information

Supplementary Table S1

Supplementary Table S2

Supplementary Table S3
