# Supplementary material for: WRINKLED1 transcription factor orchestrates the regulation of carbon partitioning for C18:1 (oleic acid) accumulation in Siberian apricot kernel
Source: Sci Rep. 2019 Feb 25;9:2693. doi: 10.1038/s41598-019-39236-9 (PMC6389899; doi:10.1038/s41598-019-39236-9)
Supplement: Supplementary file 1 — Supplementary information [file 41598_2019_39236_MOESM1_ESM.docx]

***WRINKLED1* transcription factor** **orchestrates the regulation of carbon partitioning for C18:1 (oleic acid) accumulation in Siberian apricot kernel**

Shuya Deng^1†^, Yiting Mai^1†^, Lanya Shui^1^, Jun Niu^1*^

^1^ Hainan Key Laboratory for Sustainable Utilization of Tropical Bioresource, Institute of Tropical Agriculture and Forestry, Hainan University, Haikou, Hainan 570228, China

†Equal contributors

* Corresponding author: +86-898-66268107; [niujun5555@163.com](mailto:niujun5555@163.com)

| Authors | Email addresses |
| --- | --- |
| Shuya Deng | dddssy1125@163.com |
| Yiting Mai | m18289620582@163.com |
| Lanya Shui | Shuilanya123@163.com |
| Jun Niu | niujun5555@163.com |

**Supplementary Table S1 Annotation and expression in oil palm, rapessed, castor, burning bush and siberian apricot.** The expression levels of genes were estimated using FPKM. Nucleotide sequence information can be found with corresponding AT locus ID.

**Supplementary Table S2 List of genes that are correlated with WRI1 in our analysis.** Using the R package of WGCNA, automatic construction of the gene network and identification of modules were conducted according to a convenient one-step network construction. Additionally, WRI1-coexpressed genes were used for Metascape analysis.

**Supplementary Table S3 Analysis of *cis*-acting elements of three gene promoter.** Red word represents the AW-box.

**Supplementary Table S4 Primer sequences used for promoter clone and qRT-PCR.**

|  | **Genes** | **Forward primer** | | **Reverse primer** |
| --- | --- | --- | --- | --- |
| **Promoter clone** | *PEPCK* | CCAATCAAGTACTGGGCCTCACATC | CTCGCCGTTCCCGTTCGAC | |
|  | *BS* | ACTATGGGAACCTGGACAAATGGTG | AGACTGACATTTTTGGGCTTCGCT | |
|  | *SAD* | TTATTCAAAACTGCTCTATTCACCA | GAGGTTGTGAGATGGGAAGGT | |
| **qRT-PCR analysis** | *GUS* | ATCCGGTCAGTGGCAGTGAAGG | CAGCGTAAGGGTAATGCGAG | |
|  | *WRI1* | CAACCCTGAATTTCCCGATAGA | GACGCAATGAGGCTAGGTATT | |
|  | *pPK* | AGATGCAATGGAGAAGGAAGAG | CCGCTGAAGTACACCAGAAA | |
|  | *PEPCK* | GTCGGTCCACATCCAAAGAA | CATGGTCTGTGCCAGGTTTA | |
|  | *E1-α* | CAATGTGCTTGGTGGGTTTG | TCAGCACTTCCCTCCTATATCT | |
|  | *E2* | CAACGGATGCCTCTTGTCTATC | CCCGCCAAAGCTAACTGATTA | |
|  | *TAL* | GGCCACCTCCAAATGTATGA | CGAAAGCCAAACCAGTATTTCC | |
|  | *BS* | AGCAGTGTACTCTCCTCTCTATC | GGCCTTGAGTCCTGTGTTATAC | |
|  | *MCMT* | TGTGACGCAGCCAATCAA | CTCCTTTCACACCACCAGATAC | |
|  | *BC* | CCAGGAAGAATAACCGCCTAC | GGGAGTCATAGTTTGGAGGAAC | |
|  | *HAD* | GGCTCTCGTGACACTTTCTT | CTTGCCTTCCATCTTTGCTATTC | |
|  | *KASI* | GTCTGCGTTGCTTGGTATTG | GCGGCGTAGAAACAGTAATTTG | |
|  | *EAR1* | GCTGGGAGAAGAAAGGGAATTA | GTAGAGGTGCATTGGCTGAT | |
|  | *KAR* | CTGGAGCTTCTAGAGGAATTGG | CCTTAGATGACCTTGCGTAGTT | |
|  | *SAD* | GAAGAAGCCCTACCAACATACC | GAAGAAGGTCACCATGCCTATT | |
|  | *FAD2* | CTGACTGGTGTTTGGGTGATAG | AGGGCAGAGTGAAGGATTAGA | |
|  | *LPCAT1* | GCACCTGGCTACGTCATTAT | GGATAGAGTCCATGCCAAACA | |
|  | *CYP* | CAACGGATCTCAGTTCTTCGTCTGC | GACCCAACCTTCTCGATGTTCTTCA | |
|  | *UBC* | GAGACCAGCAATAACCGTGAA | TCTTGTACTCCGTGGCATCCT | |
|  | Actin | TGGAACAGGA ATGGTCAAGG | AGGGAATACAGCTCGTGGAG | |
|  | Ubiquitin | TGACTGGGAA GACCATCACC | CCTTGTCCTGGATCTTAG CTTTT | |
